# Laparoscopic paraduodenal hernia repair with bioabsorbable mesh: A case of a novel technique for a rare cause of bowel obstruction^☆^

**DOI:** 10.1016/j.ijscr.2020.03.035

**Published:** 2020-04-04

**Authors:** Bianca Kwan, Jane E. Theodore, Jason Wong

**Affiliations:** Department of Surgery, Redcliffe Hospital, Anzac Avenue, Redcliffe, QLD, Australia

**Keywords:** Paraduodenal hernia, Bioabsorbable mesh, Paraduodenal hernia repair, Internal hernia, Case report

## Abstract

**Introduction:**

Paraduodenal hernias are the most common type of internal hernia but a rare cause of bowel obstruction. Given the high risk of incarceration, obstruction and associated mortality, surgical repair is recommended.

**Presentation of case:**

We present a novel technique for laparoscopic repair of paraduodenal hernia using biosynthetic mesh to reinforce suture closure of the hernia defect. An 18-year-old healthy female presented with symptoms of recurrent subacute small bowel obstruction and right paraduodenal hernia was diagnosed on computed tomography (CT). Laparoscopic repair was performed with reinforcement of suture repair with GORE® BIO-A® Hiatal Tissue Reinforcement to decrease the risk of recurrence. The patient had an uneventful recovery and was discharged on the second post-operative day. Subsequent follow-up revealed complete resolution of symptoms.

**Discussion:**

Biosynthetic tissue reinforcement carries a lower theoretical risk of erosion than permanent mesh. A hiatal-shaped prosthesis represents a suitable shape for paraduodenal hernia repair.

**Conclusion:**

We present the first reported use of bioabsorbable mesh to repair paraduodenal hernia. This technique may minimise risk of mesh erosion and can be considered a safe and effective approach where suture repair is inadequate due to large defect size.

## Highlights

• Paraduodenal hernias are the commonest type of internal hernia.
• Suture repair of paraduodenal hernia can be reinforced using bioabsorbable mesh.
• Mesh used in hiatus hernia repair is an ideal shape for paraduodenal hernia repair.
• Laparoscopic bioabsorbable mesh repair of paraduodenal hernia is safe and effective.

## 1 Introduction

Paraduodenal hernias are the most common type of internal hernia and are responsible for 53% of all internal hernias [1]. Paraduodenal hernias are congenital, resulting from abnormal midgut rotation and retroperitoneal fixation of the bowel mesentery, leading to the protrusion of small bowel posterior to the mesocolon into paraduodenal recesses. Left-sided paraduodenal hernias are more common, making up 75% of cases [1]. Patients can present with a spectrum of symptoms, from non-specific abdominal symptoms to small bowel obstruction and ischaemia [2,3].

We present a novel technique in laparoscopic paraduodenal hernia repair, using a bioabsorbable hiatal mesh to reinforce suture closure of the hernia defect. This case is reported in line with the SCARE criteria [4].

## 2 Presentation of case

### 2.1 Case history

An 18-year-old female with no past medical or surgical history presented to the Emergency Department with 6-week history of intermittent central, colicky abdominal pain, vomiting and weight loss. Examination revealed distended abdomen with right upper quadrant tenderness.

**Fig. 1:**
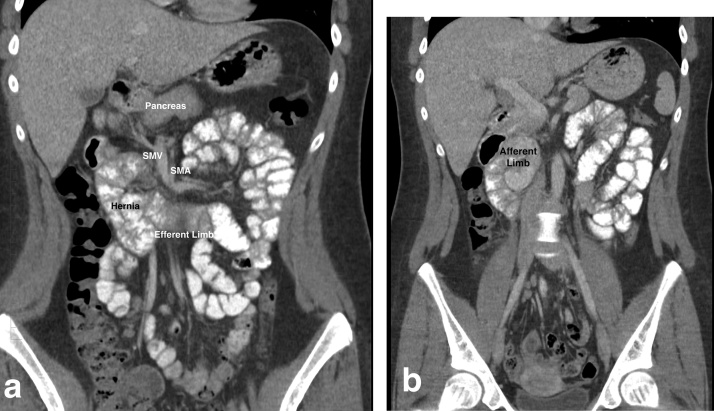
CT Images of right paraduodenal hernia. **(a)**; Right paraduodenal hernia showing sac like mass of small bowel loops to right of midline and efferent limb crossing back over to left side of abdomen. **(b)**; Afferent limb of proximal jejunum in upper right abdomen. SMV, superior mesenteric vein; SMA, superior mesenteric vein.

Computed tomography (CT) with oral and intravenous contrast showed a sac-like mass of small bowel loops in the right mid-abdomen, inferior and to the right of the duodenum. The third portion of the duodenum was oriented more craniocaudally than horizontally and the duodenojejunal flexure was to the right of the midline, suggesting duodenal malrotation. The proximal jejunum crossed the midline behind the superior mesenteric vein (SMV), pushing the SMV anteromedially. The afferent loop was narrowed and deep to the root of the small bowel mesentery. Some SMV tributaries looped posteriorly and to the right instead of their usual course. Ileal branches could be seen originating from the right instead of the left of the superior mesenteric artery (SMA). Some small bowel loops appeared dilated, however there were no signs of obstruction or volvulus. These findings were all suggestive of a right paraduodenal hernia (Fig. 1a and b).

Initial treatment involved analgesia, antiemetics, intravenous crystalloid fluids and nasogastric tube decompression.

### 2.2 Operative techniques

Optical entry in the lateral left upper quadrant was gained with 5 mm trocar and 0-degree 5 mm laparoscope. Further 12 mm left upper quadrant camera port, 12 mm umbilical and 5 mm right upper quadrant ports were placed. The patient was placed in reverse Trendelenburg position.

**Fig. 2:**
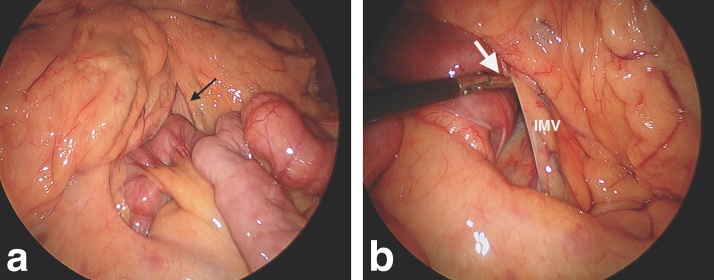
Intraoperative photographs of hernia reduction. **(a)**; Small bowel being reduced from hernia orifice (black arrow). **(b)**; Laparoscopic instrument (white arrow) inside hernia orifice, which extends superiorly and to the right. IMV, Inferior mesenteric vein.

The omentum and transverse mesocolon were displaced cephalad, exposing the infracolic compartment. This revealed small bowel herniating into a space between the duodenojejunal flexure and inferior mesenteric vein (IMV), extending behind the ascending and transverse mesocolon. 30 cm of jejunum was gently reduced from Waldeyer’s fossa (Fig. 2a and b). With no evidence of intestinal ischaemia, resection was not required. There was no evidence of intestinal malrotation, with the caecum in the correct anatomical position and no Ladd’s bands.

**Fig. 3:**
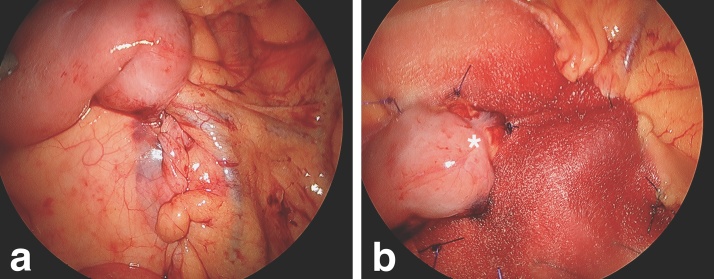
Intraoperative photographs of hernia repair. **(a)**; To close the hernia defect, the mesenteric fat lateral to the IMV was plicated over the top of the IMV and opposed to the small bowel and peritoneum at the duodenojejunal flexure with a running non-absorbable suture. **(b)**; Suture repair was reinforced with a bioabsorbable hiatal mesh, with the duodenojejunal flexure sited within the concave aspect of the mesh (asterisk). The edges of the implant were secured to mesenteric fat with interrupted and continuous absorbable sutures.

To close the 30–40 mm defect, the mesenteric fat lateral to the IMV was plicated over the top of the IMV and opposed to the small bowel and peritoneum at the duodenojejunal flexure with a running non-absorbable V-Loc suture (Covidien, Dublin, Ireland) taking care not to injure the IMV (Fig. 3a).

Suture repair did not appear adequate due to large defect size and underlying hernia cavity. The peritoneum sutured to close the defect was very thin and under some mild tension. Consequently, reinforcement lateral to the duodenojejunal flexure was performed with Gore® BIO-A® Hiatal Tissue Reinforcement (GORE, Newark, DE, USA), with the duodenojejunal flexure sited within the concave aspect of the mesh. The edges of the implant were secured to mesenteric fat with interrupted and continuous absorbable sutures (Fig. 3b).

### 2.3 Outcomes

There were no complications intraoperatively. The patient was discharged on the second postoperative day after an uneventful recovery.

At 6-week, 6-month and 1-year follow-up, there was complete resolution of symptoms. Repeat CT at 6 months revealed no recurrence of paraduodenal hernia.

## 3 Discussion

Paraduodenal hernias can manifest in a variety of presentations. Some describe chronic pain, nausea and vomiting, which resolve between episodes due to spontaneous reduction [2,3], as described in this case. More commonly, patients present with acute small bowel obstruction [5]. Physical examination may reveal a tender mass in large hernias, but is often equivocal [3,5,6]. Signs of peritonism are often absent as the hernia is retroperitoneal [7].

Radiological investigations are useful, as history and examination findings are often vague. CT may show a well-circumscribed cluster of small bowel loops to the left or right of the midline [6,8]. Stretching or displacement of mesenteric vessels at the entrance to the hernia orifice are another clue [1].

Left paraduodenal hernias occur through Landzert’s fossa, present in 2% of the population, situated to the left of the fourth part of the duodenum. The left colic artery (LCA) and IMV form the lateral border of this fossa and the anterior free edge of the hernia neck [6,9]. The sac lies in the descending and left transverse mesocolon. CT reveals small bowel in the left of the abdomen, lateral to the ligament of Treitz, with the IMV displaced anteriorly in the hernia neck. Mass effect can cause anterior, inferior or inferomedial displacement of the stomach, transverse colon and duodenojejunal flexure respectively [1].

Right paraduodenal hernias are caused by herniation through Waldeyer’s fossa, or the mesentericoparietal fossa [10], present in 1% of the population [1]. This is formed by the first part of the jejunal mesentery, lateral and inferior to the third part of the duodenum. The sac lies behind the ascending and transverse mesocolon, inferior to the horizontal part of the duodenum [8]. These hernias tend to be larger and more fixed than their left-sided counterparts [8]. CT shows a cluster of small bowel inferolateral to the second and third part of the duodenum. The SMA, ileocolic artery and right colic vein can be seen in the hernia neck, displaced anteriorly. Occasionally, small bowel can be seen looping around the superior mesenteric vessels at the root of the mesentery [1].

Surgical repair of paraduodenal hernias is recommended, including elective repair of asymptomatic cases. This is because up to 50% of cases will result in obstruction and a mortality rate of greater than 20% [11]. Laparoscopic repair was first described by Uematsu et al., in 1998 [12]. This is now the standard approach, leading to shorter hospital stay, earlier upgrade of diet, reduced rates of ileus [10] and less post-operative pain [11].

Repair traditionally consists of reducing hernia contents and closing the defect with sutures. Reduction may be facilitated by widening the defect, however care must be taken to avoid mesenteric vessel injury at the free edge of the hernia orifice. Some suggest ligation of these vessels [2]. If the defect is unable to be closed, widening it to make it continuous with the peritoneal cavity can be considered [12,13]. Incising the peritoneal attachments lateral to the ascending colon may be required to repair large right paraduodenal hernias. The hernia contents are then delivered through this incision and the right colon rotated over to the left side of the abdomen, mimicking the embryological positions of the pre- and post-arterial midgut segments after the first stage of rotation [3]. Other authors have also suggested this approach is appropriate where reduction is difficult due to adhesions or the hernia orifice is unable to be definitely identified [14].

Only one other case has reported use of mesh in repair of paraduodenal hernia. Palanivela et al. used a GORE-TEX® permanent, nonabsorbable mesh secured with 2–0 propylene sutures for recurrence of a left paraduodenal hernia. The authors suggested mesh could be used for large defects and recommended decision based on surgeon experience [15].

There are well-recognised risks with permanent, non-absorbable mesh, such as extrusion, erosion, infection and adhesions [16,17]. GORE® BIO-A® is completely absorbed in approximately 6 months and is broken down via hydrolysis and enzymatic degradation [16]. It is thought to present lower risk of mesh erosion while maintaining advantage of mesh reinforcement [17]. The use of biosynthetic mesh is well-established in ventral hernia repairs [16] and in reinforcement of crural closure in hiatal hernia repairs as opposed to permanent implants. Recently, some authors have also reported its use in suture and fibrin glue fixation in Peterson’s space to prevent internal hernia following Roux-en-Y gastric bypass [18].

To our knowledge, this is the first case of paraduodenal hernia repair using a biosynthetic, absorbable mesh. The decision to reinforce the suture repair with this method was based on use of this mesh in other clinical situations. The Gore® BIO-A® Hiatal Tissue Reinforcement, which was used in this case, is a U-shaped hiatal mesh usually used in hiatus hernia repairs. This has the ideal shape for repair of a paraduodenal hernia. The concave aspect of the mesh fit well around the duodenojejunal flexure, while still allowing the prosthesis to reinforce the suture closure of the defect with wide coverage.

We suggest biosynthetic absorbable mesh can be used during initial repair of paraduodenal hernias to reinforce suture line where the defect is large or there is concern over adequacy of suture repair. A hiatal U-shaped mesh represents an ideal shape for the prosthesis. This should be based on surgeon experience and familiarity with these materials. While repair was done laparoscopically in this case, it could also be used in open procedures. Using bioabsorbable mesh should reduce risk of hernia recurrence whilst circumventing the feared complications of mesh erosion associated with permanent implants.

## 4 Conclusion

Paraduodenal hernias are a rare but important cause of intestinal obstruction. We present a novel technique of using biosynthetic, absorbable mesh to reinforce suture repair of a paraduodenal hernia defect. This may minimise risk of mesh erosion and can be considered a safe and effective approach where suture repair is inadequate due to large defect size.

## Conflicts of interest

No conflict of interest exists.

## Sources of funding

No sources of funding.

## Ethical approval

We have reported a single case, not a clinical study, with no requirement for ethical approval.

## Consent

Written informed consent was obtained from the patient for publication of this case report and accompanying images. A copy of the written consent is available for review by the Editor-in-Chief of this journal on request.

## Author contribution

Dr Bianca Kwan: Investigation, Writing – original draft, Writing – Review and Editing, Visualisation.

Dr Jason Wong: Conceptualization, Writing – Review and Editing, Supervision.

Dr Jane Theodore: Writing – Review and Editing, Supervision.

## Registration of research studies

Not applicable.

## Guarantor

Dr Bianca Kwan.

## Provenance and peer review

Not commissioned, externally peer-reviewed.
